# Reexamination of Accelerometer Calibration with Energy Expenditure as Criterion: VO_2net_ Instead of MET for Age-Equivalent Physical Activity Intensity

**DOI:** 10.3390/s19153377

**Published:** 2019-08-01

**Authors:** Daniel Arvidsson, Jonatan Fridolfsson, Christoph Buck, Örjan Ekblom, Elin Ekblom-Bak, Lauren Lissner, Monica Hunsberger, Mats Börjesson

**Affiliations:** 1 Center for Health and Performance, Department of Food and Nutrition, and Sport Science, University of Gothenburg, 40530 Gothenburg, Sweden; 2 Department of Biometry and Data Management, Leibniz Institute for Prevention Research and Epidemiology—BIPS, 28359 Bremen, Germany; 3 Åstrand Laboratory of Work Physiology, Swedish School of Sport and Health Sciences, 11486 Stockholm, Sweden; 4 Department of Public Health and Community Medicine, Sahlgrenska Academy, University of Gothenburg, 40530 Gothenburg, Sweden; 5 Institute of Neuroscience and Physiology, Sahlgrenska Academy, University of Gothenburg, 40530 Gothenburg, Sweden; 6 Sahlgrenska University Hospital/Östra, 41345 Gothenburg, Sweden

**Keywords:** physical activity, accelerometer, VO_2_, calibration, MET, VO_2net_, speed, equivalent speed, free-living, children, adolescents, adults

## Abstract

Accelerometer calibration for physical activity (PA) intensity is commonly performed using Metabolic Equivalent of Task (MET) as criterion. However, MET is not an age-equivalent measure of PA intensity, which limits the use of MET-calibrated accelerometers for age-related PA investigations. We investigated calibration using VO_2net_ (VO_2gross_ − VO_2stand_; mL⋅min^−1^⋅kg^−1^) as criterion compared to MET (VO_2gross_/VO_2rest_) and the effect on assessment of free-living PA in children, adolescents and adults. Oxygen consumption and hip/thigh accelerometer data were collected during rest, stand and treadmill walk and run. Equivalent speed (Speed_eq_) was used as indicator of the absolute speed (Speed_abs_) performed with the same effort in individuals of different body size/age. The results showed that VO_2net_ was higher in younger age-groups for Speed_abs_, but was similar in the three age-groups for Speed_eq_. MET was lower in younger age-groups for both Speed_abs_ and Speed_eq_. The same VO_2net_-values respective MET-values were applied to all age-groups to develop accelerometer PA intensity cut-points. Free-living moderate-and-vigorous PA was 216, 115, 74 and 71 min/d in children, adolescents, younger and older adults with VO_2net_-calibration, but 140, 83, 74 and 41 min/d with MET-calibration, respectively. In conclusion, VO_2net_ calibration of accelerometers may provide age-equivalent measures of PA intensity/effort for more accurate age-related investigations of PA in epidemiological research.

## 1. Introduction

The use of accelerometers to measure physical activity (PA) in research have expanded tremendously since the millennium shift. An essential component of PA is the intensity performed, as PA intensity is related to the health benefits achieved [1]. Therefore, it is crucial that accelerometers provide accurate measures of PA intensity, which commonly involves determining time spent in different intensity levels such as light (LPA), moderate (MPA) and vigorous (VPA) physical activity. These PA intensity measures have been used in cross-sectional and longitudinal epidemiological studies to investigate, for example, the age-related decline in PA from childhood into adulthood and associated factors, in an attempt to identify risk groups for future cardiometabolic disease [2,3,4].

Researchers working with development of PA intensity measures from accelerometers have mainly used energy expenditure determined from measured oxygen consumption as criterion method. The most common criterion measure of absolute PA intensity is Metabolic Equivalent of Task (MET), calculated as the quotient of gross energy expenditure and resting energy expenditure. However, MET is not directly comparable between children and adults, as the contribution of resting energy expenditure to gross energy expenditure changes from childhood into adulthood [5]. The resting energy expenditure decreases from about 2.0 kcal⋅kg^−1^⋅h^−1^ at the age of 5 years to 1.0 kcal⋅kg^−1^⋅h^−1^ at the age of 18 years. When children 8–12 and adolescents 15–18 years old walk at a speed of 5.6 km⋅h^−1^ they reach a MET-value of 4.3 and 4.5, respectively, while running at 8.0 km⋅h^−1^ contributes to a MET-value of 6.7 and 8.1, respectively [6]. When adults walk and run at these speeds, they reach the MET-value of about 5.0 and 9.0, respectively [7]. Importantly, despite their lower MET-values, the younger individuals would actually exert higher degree of effort with higher oxygen consumption per kg body weight compared to the older individuals [8,9] as they are moving with higher step frequency [10,11].

It is common in calibration of accelerometers to use 3.0 and 6.0 METs as cut-points for MPA and VPA, respectively, in both children and adults [12], but even higher values have been implemented in children, i.e., 4.0 and 7.0 METs [2,3,13], with the argument that it adjusts for their higher resting energy expenditure. In fact, we put higher requirements on children than on adults to achieve MPA and VPA. Therefore, the current calibration procedure for absolute PA intensity using MET cannot be used to compare the PA in different age-groups. Theoretically, this may contribute to underestimation of the PA in children relative to adults and incorrect conclusions concerning the decline in PA from childhood into adulthood and identification of individuals being physically inactive.

Attempts have been made to identify alternative criterion measures of activity intensity from oxygen uptake [14]. One such measure is the mass-specific net oxygen consumption (VO_2net_, mL⋅kg^−1^⋅min^−1^), subtracting resting oxygen uptake (VO_2rest_) from gross oxygen uptake (VO_2gross_). However, this measure is not equivalent by age, as it decreases from childhood into adulthood [14]. In biomechanical research VO_2net_ is instead calculated by subtracting standing oxygen uptake (VO_2stand_) from VO_2gross_ [10,11]. This definition considers the energy requirement of dynamic movement only and therefore matches the dynamic acceleration captured by accelerometers. The definition of subtracting VO_2rest_ from VO_2gross_ would also include oxygen consumption for muscular support during movement which is not captured by the accelerometer. The literature has not clearly demonstrated whether VO_2net_ (VO_2gross_ − VO_2stand_) as a measure of activity intensity is equivalent by age [8,9,10,11,15]. It would make sense that children have higher VO_2net_ than adults at the same absolute speed (Speed_abs_) [8,9], as the they are moving with a higher degree of effort due to the higher step frequency [10,11]. Instead, the VO_2net_ is more similar at the kinematically equivalent speed (Speed_eq_) [8,9,15], which reflects the Speed_abs_ two individuals of different body size are moving at with the same degree of effort, i.e., shorter individuals would move at a slower Speed_abs_ than taller individuals. Therefore, further investigations of the VO_2net_ as a measure of activity intensity equivalent by age is warranted, relating it to the Speed_eq_ to determine whether it reflects a similar effort performed by individuals of different body size and age. If it does, it can be used to calibrate accelerometer PA intensity measures equivalent by age to be implemented into epidemiological research for age-related investigations of PA.

The ActiGraph counts has been the most common accelerometer-based measure of PA activity intensity in research, mostly from hip recordings [12]. However, the processing of raw acceleration to counts demonstrates a measurement error of increasing attenuation of the counts with increasing PA intensity, which is greater in children than in adults [16,17]. This error was explained by the use of a too narrow band-pass filter. When the filter was expanded to a more optimal level, children and adults produced similar accelerometer outcomes from hip recordings for the same absolute movement speed. This indicates that an accelerometer placed at the hip would capture total mechanical work in both children and adults, which would be identical at the same Speed_abs_ [11,17]. The measurement error with the ActiGraph counts may have contributed to the confusion of the METs in the calibration of accelerometers, where children demonstrated both lower METs and lower counts than adults at the same Speed_abs_. In fact, children should have demonstrated lower METs but similar counts at the same Speed_abs_. These different outcomes would have contributed to different calibration equations and cut-points for PA intensity. While the comparability of accelerometer measures of PA intensity by age has been investigated for the hip placement, less is known about the thigh placement [17,18,19]. The thigh placement offers additional possibilities for assessment of sedentary time (sitting, standing), biking (i.e., cadence) as well as non-wear time.

Therefore, the aim of the present study was to investigate the calibration of hip and thigh accelerometers in children and adults using VO_2net_ (VO_2gross_ − VO_2stand_) as criterion for absolute PA intensity and the effect on assessment of free-living PA. With the application of a novel calibration procedure, this study may contribute to improve the assessment of PA intensity in children and to establish a measure of PA intensity equivalent by age.

## 2. Materials and Methods

### 2.1. Study Design

Two sub-studies were performed:

1. A calibration study including sit, stand, walk and run on a treadmill wearing accelerometers at the hip and thigh and with measured oxygen consumption to develop accelerometer cut-points for PA intensity.
2. Investigation of free-living PA in children, adolescents and adults comparing application of VO_2net_ (VO_2gross_ − VO_2stand_) cut-points to standard MET cut-points.

### 2.2. Samples

Participants in the first sub-study were 10 children 9–11 years old, 10 adolescents 14–16 years old and 10 adults 23–44 years old. They were recruited among staff and students and their children by written and verbal information at the Department of Food and Nutrition, and Sport Science in Sweden and among their friends and children [17]. All participants provided informed consent and the protocol was approved by the ethical committee at the University of Gothenburg.

The second sub-study used Swedish data collected in the European I.Family study (EC: FP7, No. 266044) [20], which is an extension of the IDEFICS study (EC: FP6, No. 016181) investigating children’s health, as well as data collected in the LIV2013 study of adult’s health in Sweden [21]. The I.Family sample consisted of 417 Swedish children and adolescents (50% female) with a mean (SD) age of 10.9 (2.4) years, the range being 4–16 years. The LIV2013 study consisted of 735 Swedish adults (24% female) with a mean (SD) age of 48.4 years (11.6), the range being 21–67 years. The participants in these studies have provided informed consents and the studies have been approved by the ethical committees.

### 2.3. Protocols, Measures and Data Analysis

#### 2.3.1. Calibration Study

The calibration study was performed in the biomechanical–physiological lab at the Center for Health and Performance, Gothenburg, Sweden. The participants were asked to refrain from food intake and strenuous PA 3–4 h prior to measurements. Body weight and height were measured and one Axivity AX3 triaxial accelerometer (Axivity Ltd., Newcastle upon Tyne, UK) was attached over the right hip in an elastic belt around the waist and another at the middle of the front side of the right thigh using medical tape. The accelerometers were set to record data at a sample rate of 100 Hz and an acceleration range of ±8 g and the raw triaxial data was extracted with the OmGUI software (Axivity Ltd., Newcastle upon Tyne, UK). Oxygen consumption was measured using the stationary metabolic system Oxycon Pro (Jaeger, BD Corporation, Franklin Lakes, NJ, USA) where the participants breath through a face-mask with a turbine flow meter and expired air-sampler. Oxycon Pro has shown high accuracy when evaluated with the Douglas bag method [22]. The activities included consisted of seated rest in an armchair for 20 min to determine resting energy expenditure (REE) followed by 4 min standing, walking at 3, 4, 5 and 6 km⋅h^−1^ and running at 8 and 10 km⋅h^−1^ on a treadmill during 4 min at each speed without interruption, in order to reach steady-state [23]. VO_2_ data was collected during all 20 min and respective 4 min activities.

The triaxial acceleration data were processed using our new algorithm to produce a measure of PA intensity expressed in mg, including a band-pass filter with the cut-point range of 0.29–10 Hz that was shown to include all relevant information to assess activity intensity and to minimize inclusion of noise [17,18]. However, acceleration data was down-sampled to 30 Hz and truncated to ±6 g before processing to match the corresponding ranges of the ActiGraph GT3X+ accelerometer used in the free-living measurements. VO_2_ data from the 20 min of resting was filtered with a moving average filter with a window size of 2 min and the minimum value was considered the individual REE. One minute of data (accelerometer, VO_2_) captured between duration 2:45 and 3:45 at standing and at each treadmill speed was used for calibration. VO_2net_ was calculated by subtracting VO_2stand_ from VO_2gross_ divided by body weight (mL⋅min^−1^⋅kg^−1^), while MET-values were calculated by the quotient of the VO_2gross_ and VO_2rest_.

In each age-group, the relationships between Speed_abs_ and accelerometer output (mg), Speed_abs_ and VO_2net_ and Speed_abs_ and MET were investigated, as well as the relationship between Speed_eq_ and VO_2net_ and Speed_eq_ and MET. The Speed_eq_ was calculated as Speed_eq_ = V^2^⋅g^−1^⋅h^−1^ (V = Speed_abs_ (m⋅s^−1^), g = gravity (9.81 m⋅s^−2^), h = body height (m)), and represent the Speed_abs_ performed with similar kinematical effort in individuals of different body size [8,9,15]. Smoothing splines were fitted to the data from each age group, with the intercept forced to zero VO_2net_ or 1 MET, and zero acceleration. Based on previous biomechanical and physiological research, we made the following assumptions:

- The three age-groups fall on the same regression line between Speed_abs_ and hip accelerometer output, as the hip accelerometer represents total mechanical work which would be the same for the same Speed_abs_ in the three age-groups [10,11,17].
- The three age-groups would have less similarity in the regression line between Speed_abs_ and thigh accelerometer output, as the mechanical work of moving the limbs is higher in shorter than taller individuals [10,11,17].
- The three age-groups would fall on the same regression line between Speed_eq_ and VO_2net_, if VO_2net_ represent the same effort in the age-groups.
- The three age-groups would fall on different regression lines between Speed_eq_ and MET, if MET does not represent the same effort in the age-groups.
- The three age-groups would fall on different regression lines between VO_2net_ and accelerometer output, if the same VO_2net_ in the age-groups would mean the same Speed_eq_ and therefore the same effort, but at the same time lower Speed_abs_ in shorter individuals and correspondingly lower mechanical work and accelerometer output.

MET cut-points at 1.5, 3.0, 6.0 and 9.0 were implemented to represent LPA, MPA, VPA and very vigorous PA (VVPA), respectively, which is in line with previous literature [12]. To calibrate accelerometer data to VO_2net_, the first step was to determine the VO_2net_ corresponding to 3.0, 6.0 and 9.0 METs in adults by linear regression. The next step was to apply the same VO_2net_ cut-points (and therefore the same effort) to all three age-groups to calibrate the corresponding accelerometer mg cut-points based on fitted smoothing splines. For comparison, accelerometer mg cut-points were also determined using the traditional 1.5, 3.0, 6.0 and 9.0 METs in all three age-groups.

#### 2.3.2. Free-Living Study

In both the I.Family study and the LIV2013 study the ActiGraph GT3X+ (ActiGraph, Pensacola, FL, USA) was used to collect acceleration data and was worn over the right hip in an elastic belt around the waist. The participants were instructed to wear the accelerometer for seven days and to remove it during sleep and during water-based activities. The accelerometer was set to record acceleration data at 30 Hz sampling rate with an acceleration range of ±6 g and idle sleep mode were enabled. The raw triaxial data was extracted according to available file specifications [24].

Raw triaxial data was processed using our new algorithm to produce a measure of PA intensity expressed in mg including a band-pass filter with the cut-point range of 0.29–10 Hz [17,18]. The epoch-length was set to 3 s to capture children’s activities as well as intermittent activities in adults [18]. Valid days with at least 12 h were included in the analyses. Samples in which the sensor status was idle according to the idle sleep-mode were considered non-wear time. More subjects in the adult group wore the accelerometer during sleep compared to the children; therefore, the acceleration recorded between 23:00–06:00 was not included in the analysis. Our previous research has confirmed that processing of GT3X+ data and AX3 data generate similar output [16].

Accelerometer mg cut-points for LPA, MPA, VPA and VVPA from the VO_2net_-based calibration and the standard MET-based calibration were applied. The participants were divided into four age-categories: Children (<13 years, n = 321 (50% female), adolescents (13–16 years, n = 96 (50% females)), younger adults (<50 years, n = 366 (75% female)) and older adults (≥50 years, n = 369 (68% female)). The time distribution in the intensity categories was compared between the age-categories with the VO_2net_-based calibration versus the MET-based calibration. As the VO_2net_-based calibration would imply lower Speed_abs_ and mechanical work in children and adolescents compared to the MET-based calibration, and consequently lower accelerometer mg cut-points, we assume more free-living PA in these age groups applying the VO_2net_-based accelerometer mg cut-points.

All data processing and analyses in the two sub-studies were performed in MATLAB 2018b (MathWorks, Natick, MA, USA).

## 3. Results

### 3.1. Calibration Study

**Figure 1:**
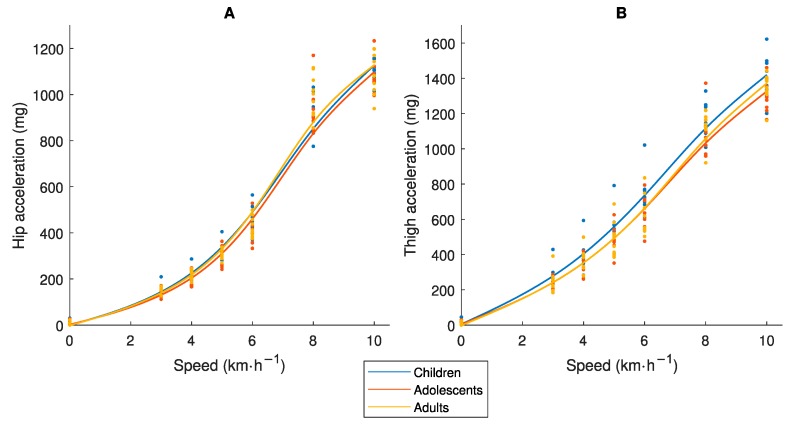
Relationship between absolute speed (Speed_abs_) and the accelerometer measure of physical activity (PA) intensity (mg) for (**A**) hip and (**B**) thigh placement, in children 9–11, adolescents 14–16 and adults 23–44 years old.

Figure 1 presents the relationship between Speed_abs_ and the accelerometer output. It demonstrates that the three age-groups fall approximately on the same regression line for hip data, while children show higher values compared to the other age-groups for the thigh placement. These results confirm our assumptions of similar mechanical work captured with the hip placement and less similarity with the thigh placement.

**Figure 2:**
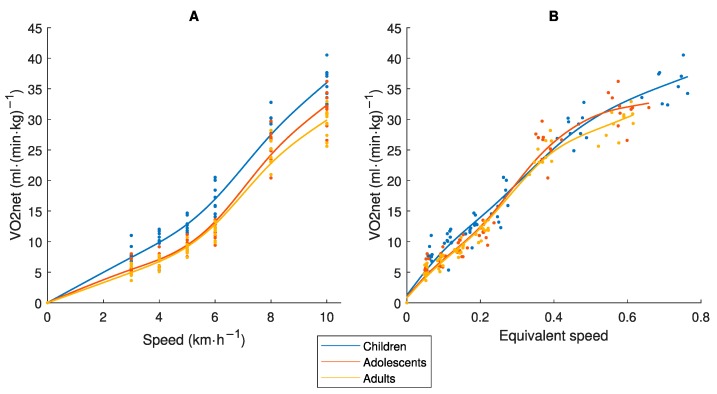
Relationship between (**A**) Speed_abs_ or (**B**) equivalent speed (Speed_eq_) and net oxygen consumption (VO_2net_), in children 9–11, adolescents 14–16 and adults 23–44 years old.

Figure 2 present the relationship between Speed_abs_ and VO_2net_ and between Speed_eq_ and VO_2net_, respectively. It shows that while children have higher VO_2net_ than the other age-groups for the same Speed_abs_, there was approximately one common regression line with the Speed_eq_. VO_2net_ may therefore be considered a measure of similar effort in different age-groups according to our assumption.

**Figure 3:**
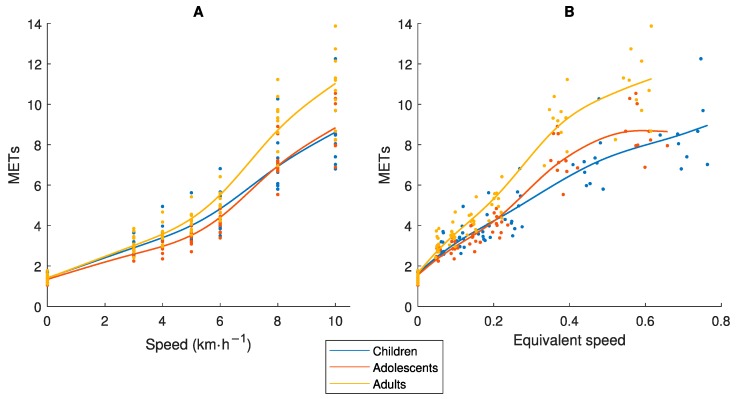
Relationship between (**A**) Speed_abs_ or (**B**) Speed_eq_ and Metabolic Equivalent of Task (MET), in children 9–11, adolescents 14–16 and adults 23–44 years old.

Figure 3 display the relationship between Speed_abs_ and MET and between Speed_eq_ and MET, respectively. The MET-values differ between the three age-groups for both Speed_abs_ and Speed_eq_, which confirms the assumption that MET is not a measure of effort comparable between age-groups.

**Figure 4:**
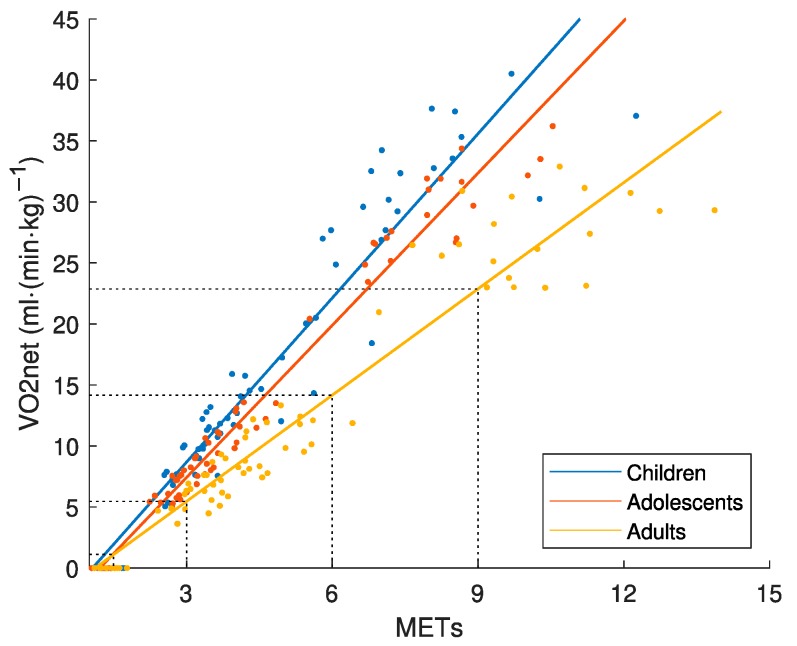
Definition of the common VO_2net_ cut-points representing light PA (LPA), moderate PA (MPA), vigorous PA (VPA) and very vigorous PA (VVPA) based on adult MET values, in children 9–11, adolescents 14–16 and adults 23–44 years old.

Figure 4 presents the relationship between MET and the corresponding VO_2net_. It is the starting-point to determine the VO_2net_ cut-points for LPA (1.5 METs), MPA (3.0 METs), VPA (6.0 METs) and VVPA (9.0 METs) based on adult data. If the same MET-values had been applied to the children and adolescent groups, considerable higher VO_2net_-values (and effort) had been used for accelerometer calibration in these age-groups.

**Figure 5:**
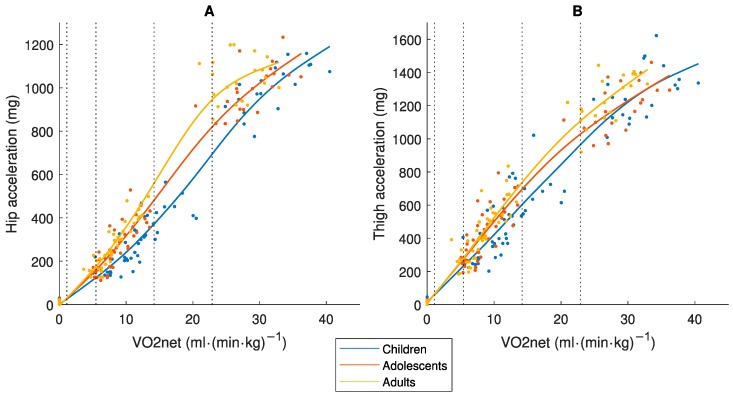
Calibration of accelerometer measures of absolute PA intensity (mg) for (**A**) hip and (**B**) thigh placement in children 9–11, adolescents 14–16 and adults 23–44 years old, using the common VO_2net_ cut-points defined and presented in Figure 4.

The next step was to apply the common VO_2net_ cut-points to all three age-groups to determine the corresponding accelerometer mg values (Figure 5). The three age-groups achieved different regression lines, with the lowest accelerometer cut-points observed in children. Hence, when moving with the same effort, the three age-groups move at different Speed_abs_ and consequently do different mechanical work. Still, there were some overlapping between the age-groups.

**Figure 6:**
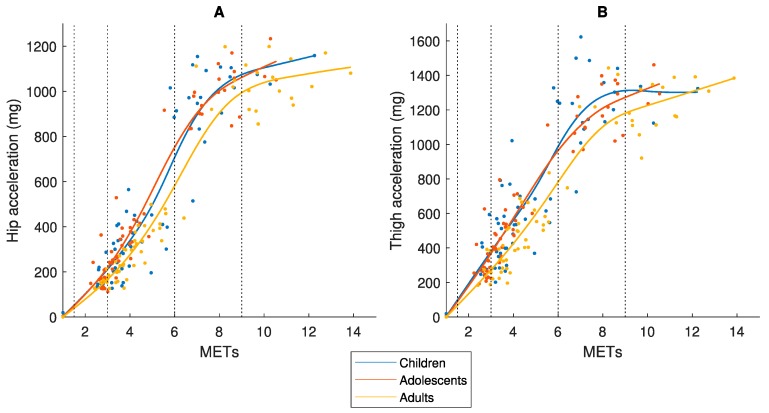
Calibration of the accelerometer measure of absolute PA intensity (mg) for (**A**) hip and (**B**) thigh placement in children 9–11, adolescents 14–16 and adults 23–44 years old, using the standard MET cut-points (1.5, 3.0, 6.0, 9.0) in all three groups.

In contrast, when MET-based cut-points were applied, the adults achieved the lowest accelerometer values (Figure 6). Table 1 provides the numerical values of the VO_2net_-based and the MET-based cut-points for LPA, MPA, VPA and VVPA for the two body placements in the three age-groups, expressed in mg.

### 3.2. Free-Living Study

The results from the free-living study shows that when applying the VO_2net_ calibration, there was a considerable increase in the time being physically active in children and adolescents compared with application of the standard MET calibration (Table 2). For example, the time spent in moderate-and-vigorous PA (MVPA = MPA + VPA + VVPA) accounted for 13.8% of the wear-time in children and 8.1% in adolescents with the MET calibration, but increased to 21.2% and 11.2%, respectively, with the VO_2net_ calibration. Consequently, the difference in MVPA between children and adolescents versus adults increases. As an example, with the standard MET calibration the difference in MVPA between children and younger adults is 6.5% units, while with the VO_2net_ calibration it is 13.9% units. In daily minutes, the difference corresponds to 66 min and 142 min, respectively.

## 4. Discussion

The novelty of this study is to propose a criterion method (VO_2net_, mL⋅kg^−1^⋅min^−1^) to calibrate accelerometer PA intensity measures equivalent by age based on data collected at the hip and at the thigh. It was applied to our improved processing of acceleration data [16,17,18,19] to generate new cut-points for LPA, MPA, VPA and VVPA in children, adolescents and adults. These outcomes were supported by the finding that the relationship between Speed_eq_ and VO_2net_ (VO_2gross_ − VO_2stand_) was similar in children, adolescents and adults, indicating that VO_2net_ is a measure of similar effort in the three age-groups, in contrast to the more commonly used MET in accelerometer calibration which was not a measure of PA intensity equivalent by age. When our new cut-points for PA intensity levels were applied to free-living acceleration data from the hip, the difference in PA between children, adolescents and adults increased compared to the application of the standard MET-based cut-points. The importance of the findings in this study is to allow the possibility to directly relate the PA intensity level between age-groups and thereby more accurately investigate the age-related changes in PA from childhood into adulthood and associated factors. A larger decline in PA from childhood into adults may be expected compared to what is anticipated from previous research.

Two individuals of different body size perform an activity with different movement patterns. A short individual takes shorter steps with smaller acceleration amplitude but at higher frequency, while the taller individual takes longer steps with larger acceleration amplitude but at lower frequency [10,11,17]. Biomechanical theory demonstrates that the total mass-specific mechanical work is the same in short and tall individuals moving at the same Speed_abs_, but that the shorter individual generates more internal work related to the higher frequency of moving the limbs while the taller individual generates more external work related to the movement of the center of mass [10,11]. Consequently, one would expect an accelerometer placed at the hip to generate higher values in the taller individual while the opposite would occur with the placement on the thigh. Interestingly, our results (Figure 1A) show that the hip placement generates similar accelerometer output for the same Speed_abs_ in children, adolescents and adults, indicating that all the acceleration signals captured represent total mechanical work. Hence, an accelerometer placed at the hip can be used to compare the total mechanical work performed between age-groups. Instead, the thigh placement generated higher accelerometer output in the children compared to the other groups (Figure 1B), indicating that this placement captures more internal work which is not comparable between age-groups. In contrast to the results herein, the original ActiGraph counts from hip recordings are lower in children than in adults for the same Speed_abs_ [25,26], while the opposite occur with the Euclidian Norm Minus One (ENMO) accelerometer output [27]. Consequently, none of these methods provide measures of mechanical work equivalent by age. In the case of the ActiGraph counts, this age difference is caused by a well-defined processing error [16]. In the case of the ENMO accelerometer output, it may be a processing error less clearly defined. With the ENMO method, all negative accelerations are set to zero after subtracting 1 g from the vector magnitude [27]. This means that acceleration signals generated with larger amplitude but at lower frequency (as in adults) will be excluded to larger extent compared to acceleration signals with a lower amplitude but at a higher frequency (as in children). In contrast, the ActiGraph counts and the accelerometer method developed in our research group are aggregations of both positive and negative accelerations.

Even if an accelerometer placed at the hip would capture total mechanical work at the same Speed_abs_, the activity is performed with different effort and energy cost in children compared to adults (Figure 2A) [8,9], and therefore has different physiological loads/health effects on the body. Consequently, an accelerometer output needs to be calibrated against a criterion measure of equivalent effort/load by age. The MET was developed to provide a criterion measure of absolute PA intensity. Our study showed that the MET was not equivalent by age as different values were achieved in children, adolescents and adults for the same Speed_abs_ and Speed_eq_ (Figure 3). If we compare the results in Figure 5 and Figure 6, we clearly see the consequence of applying the MET-based accelerometer calibration: Higher accelerometer cut-points are set for younger individuals to reach MPA, VPA and VVPA, when they are actually performing the activity at these PA intensity levels with a higher degree of effort according to the Speed_eq_ (Figure 3B) and with a higher energy cost according to the VO_2net_ (Figure 4) compared to the older individuals. This calibration error will contribute to the underestimation of the PA in children relative to adults.

Alternative criterion measures of PA intensity equivalent by age have been investigated, for example mass-specific VO_2gross_ (mL⋅kg^−1^⋅min^−1^), VO_2net_ (VO_2gross_ − VO_2rest_, mL⋅kg^−1^⋅min^−1^) and VO2_allom_ (mL⋅kg^−0.75^⋅min^−1^) [14]. None of the measures was optimal for the PA intensity range. The allometric scaling seems to work well for ambulatory activities and the MVPA intensity range [14] and has previously been proposed as an accurate criterion measures for accelerometer calibration [28]. We based our choice of VO_2net_ (VO_2gross_ − VO_2stand_, mL⋅kg^−1^⋅min^−1^) on that this measure captures the dynamic movement only and therefore matches the dynamic acceleration captured by an accelerometer. Although, previous research has shown a remaining difference by age when this measure was related to Speed_eq_. For example, at Speed_eq_ = 0.3, the VO_2net_ was about 230 and 200 mL⋅kg^−1^⋅km^−1^ in children and adults, respectively [8]. We also observed some fluctuations in the difference between the age-groups across Speed_eq_ range (Figure 2B), but the relevance of these small differences and those found in the study by McCann et al. [8] is unclear and requires further investigations. We still find our results to provide a strong indication of VO_2net_ as a criterion measure of PA activity intensity equivalent by age and suitable for accelerometer calibrations.

A limitation with the VO_2net_ measure is that there are no established PA intensity cut-points as for the MET measure (i.e., 3.0 and 6.0 METs). We based our calibration procedure on the adult MET-values and cut-points for LPA, MPA, VPA and VVPA (Figure 4), as the MET as a measure of absolute PA intensity was initially developed in adults [29], and translated them to the corresponding VO_2net_. The same VO_2net_ cut-points were thereafter applied to all three age-groups to represent PA intensity levels equivalent by age and used for the accelerometer calibration (Figure 5). As children, adolescents and adults produce the same total mechanical work at the same Speed_abs_ [10,11], which is captured by an accelerometer at the hip (Figure 1A), and as VO_2net_ differs between these age-groups at the same Speed_abs_ (Figure 2A) due to different efforts related to step frequency [10,11] but is similar at the same Speed_eq_ (Figure 2B), these age-groups will present different regression lines between VO_2net_ and the accelerometer output and consequently also different accelerometer cut-points (children < adolescents < adults). The somewhat smaller difference in the VO_2net_-acceleration regression lines between the age-groups with the thigh placement (Figure 5) is explained by differences in the accelerometer output, which is supported by the biomechanical literature showing that smaller individuals produce more internal work [10,11] and consequently more thigh acceleration. Our calibration procedure requires knowledge about speed of movement. An alternative procedure embraced in calibration and validation studies, is to let the participants perform walking and running at a self-selected pace not involving the measurement of movement speed [13]. It is possible that the self-selected paces correspond to the same Speed_eq_ in different age-groups. The accelerometer calibration research field has postulated that a variety of activity types (including intermittent) should be included in calibration protocols to provide calibration algorithms representative of people’s daily activity pattern [30], not only walking and running. Still, that protocol would include activities where the steady-state is not easily attained in order to accurately assess the oxygen consumption of an activity [23], which is the case for protocols including only continuous walking and running as in the present study.

The contribution of our study can be exemplified based on two large epidemiological studies where the PA has been compared between children and adults. Cross-sectional analyses of accelerometer data from the National Health and Nutrition Examination Survey (NHANES) have shown that boys and girls 6–11 years old spend 95 and 75 min daily in MVPA, respectively, while men and women 40–49 years old attained 35 and 19 min, respectively [2]. Longitudinal analyses of accelerometer data in the European Youth Heart Study between ages 9 and 15 years old demonstrated a decline in MVPA from 100 to 52 min daily in Swedish boys and from 73 to 44 min daily in Swedish girls [4]. In another sample in this study, there was a decline between ages 15 and 21 years old from 68 to 58 min daily in Swedish males and from 46 to 40 min daily in Swedish females. These studies used MET-based accelerometer cut-points for MPA and VPA, applying similar (3.0 and 6.0 METs) or higher (4.0, 7.0 vs. 3.0, 6.0 METs) MET-values in children compared to adults. Consequently, higher demands were required from the children to reach MPA or VPA than in the adults. Further, the acceleration data was processed to the original ActiGraph counts with its limitations to assess PA intensity [16,17,18,19]. In addition, the aggregation of the acceleration data was performed into 60 s epochs, which would be insensitive to the movement pattern of children [1]. The application of our improved processing of accelerometer data [17,18] and calibration procedure presented herein together with the use of 3 s epochs would have demonstrated a larger decline in MVPA from childhood into adulthood. Consequently, there may have been an underestimation of the change in the risk of future cardiometabolic disease in those studies.

A further complication with the widely adopted 3.0 MET cut-point is that it may have been set too low to accurately represent MPA in the general population. The American College of Sports Medicine Position Stand classify 4.8–7.1 METs and 4.0–5.9 METs as MPA in young respective middle-aged adults [31]. Further, 3.0 METs was already reached at comfortable walking by all age-groups in the present study as well as in other studies of children and adults [6,7,13,14]. Consequently, people would be considered physically active too easily. This issue requires further discussion to be settled, but a higher cut-point for MPA might be considered in investigations of the general population.

The present study has several strengths and limitations. Raw acceleration data were processed with algorithms improving assessment of PA intensity [17,18]. Further, short 3 s epochs were applied to improve assessment of children’s PA pattern [1] as well as intermittent PA. Calibration of activity intensity cut-points was performed on a treadmill with four minutes at each speed. Previous studies with other accelerometers have showed inconsistent results concerning the impact of setting (treadmill vs. ground) on accelerometer output [32,33]. Calibration performed with walking and running on a treadmill may not be applicable to other types of activities. As we have commented on earlier in the discussion section, using oxygen consumption as criterion measure of PA intensity requires steady-state of oxygen consumption data [23]. Still, the steady-state requirement may be a challenge as a large part of free-living PA does not fulfill it. The present calibration included less data points in the VPA range (see Figure 5) compared to the MPA and VVPA ranges which may affect the precision of resultant regression lines and cut-points. The calibration sample age-range of 9–44 years did not fully represent the free-living sample age-range of 4–67 years, which may affect the results of the free-living stub-study.

## 5. Conclusions

We propose VO_2net_ (VO_2gross_ − VO_2stand_, mL⋅kg^−1^⋅min^−1^), instead of MET (VO_2gross_/VO_2rest_), to be a criterion measure for accelerometer calibration in children, adolescents and adults. This conclusion is based on the finding that these age-groups showed similar relationship between Speed_eq_ and VO_2net_, and consequently VO_2net_ may be considered a measure of absolute PA intensity equivalent by age. The same similarity between the age-groups was not found for the relationship between Speed_eq_ and MET. New VO_2net_-based accelerometer cut-points were developed and when they were applied to free-living data, the difference in PA between children, adolescents and adults was increased compared to applying MET-based accelerometer cut-points. The implications of these findings are that PA can now be directly compared between age-groups in epidemiological research to investigate changes in PA behaviors from childhood into adulthood and associated factors related to risk for cardiometabolic disease. We contribute with new accelerometer cut-points for absolute PA intensity for the hip and thigh placement in children, adolescents and adults (Table 1), to be applicable to accelerometer output generated from raw triaxial acceleration data [17,18]. The algorithms for processing the raw acceleration data are available by request to the authors. Still, this is a small and explorative study and further research is needed to evaluate the robustness of the VO_2net_ calibration method proposed.

## Figures and Tables

**Table 1:** Accelerometer cut-points (mg) by body placement, age-group and calibration method.

Unit: mg	Children		Adolescents		Adults	
	VO_2net_	MET	VO_2net_	MET	VO_2net_	MET
**Hip placement**						
Light	29	52	32	50	39	39
Moderate	124	214	157	219	167	167
Vigorous	368	704	482	753	582	582
Very vigorous	695	1075	830	1062	994	994
	R^2^ = 0.96	R^2^ = 0.96	R^2^ = 0.97	R^2^ = 0.98	R^2^ = 0.98	R^2^ = 0.98
**Thigh placement**						
Light	60	99	63	90	67	67
Moderate	234	380	274	368	273	273
Vigorous	603	987	700	964	782	782
Very vigorous	964	1312	1034	1272	1181	1181
	R^2^ = 0.93	R^2^ = 0.95	R^2^ = 0.95	R^2^ = 0.98	R^2^ = 0.98	R^2^ = 0.98

Children 9–11, adolescents 14–16 and adults 23–44 years old. VO_2net_ cut-points (mL⋅min^−1^⋅kg^−1^): LPA 1.1, MPA 5.5, VPA 14.2 and VVPA 22.9. MET cut-points (METs): LPA 1.5, MPA 3.0, VPA 6.0 and VVPA 9.0. Explained variation (R^2^) is presented for the fit of the regression line between VO_2net_/MET and accelerometer mg.

**Table 2:** Time spent daily (min and % of wear time) in different intensity levels by age-group and calibration method for the hip placement.

Mean (SD)	Children (n = 321)		Adolescents (n = 96)		Adults <50 (n = 366)		Adults ≥50 (n = 369)	
Calibration	Min	%	Min	%	Min	%	Min	%
**VO_2net_**								
SED	661 (125)	64.8 (12.2)	786 (114)	77.1 (11.1)	826 (56)	81.0 (5.5)	821 (60)	80.5 (5.9)
LPA	142 (41)	14.0 (4.1)	119 (52)	11.7 (5.1)	119 (37)	11.7 (3.6)	128 (39)	12.5 (3.9)
MPA	154 (57)	15.1 (5.6)	99 (58)	9.7 (5.7)	71 (26)	7.0 (2.5)	69 (29)	6.8 (2.8)
VPA	47 (37)	4.6 (3.6)	12 (12)	1.1 (1.2)	2 (4)	0.2 (0.4)	2 (3)	0.2 (0.3)
VVPA	15 (13)	1.5 (1.3)	4 (4)	0.4 (0.4)	1 (3)	0.1 (0.3)	0 (1)	0.0 (0.1)
**MET**								
SED	710 (117)	69.6 (11.5)	814 (104)	77.8 (10.2)	826 (56)	81.0 (5.5)	821 (60)	80.5 (5.9)
LPA	170 (51)	16.7 (5.0)	123 (56)	12.0 (5.4)	119 (37)	11.7 (3.6)	128 (39)	12.5 (3.9)
MPA	125 (70)	12.3 (6.9)	78 (54)	7.6 (5.3)	71 (26)	7.0 (2.5)	69 (29)	6.8 (2.8)
VPA	10 (8)	1.0 (0.7)	3 (3)	0.3 (0.3)	2 (4)	0.2 (0.4)	2 (3)	0.2 (0.3)
VVPA	5 (6)	0.5 (0.6)	2 (2)	0.2 (0.2)	1 (3)	0.1 (0.3)	0 (1)	0.0 (0.1)

As the VO_2net_ cut-points are determined from the MET-values for the different intensity levels in adults (i.e., 1.5, 3.0, 6.0 and 9.0), there will be no difference in the accelerometer cut-points in adults applying VO_2net_ calibration vs. MET calibration. Consequently, the time spent in the different intensity levels does not differ between VO_2net_ calibration vs. MET calibration in adults. Children include the age-range 4–12 years, adolescents 13–16 years, and the adult group was divided into younger adults (<50 years, range 21–49 years) and older adults (≥50 years, range 50–67 years).
